# Level of agreement between patient-reported EQ-5D responses and EQ-5D responses mapped from the SF-12 in an injury population

**DOI:** 10.1186/s12963-015-0047-z

**Published:** 2015-06-13

**Authors:** Belinda J Gabbe, Emma McDermott, Pam M Simpson, Sarah Derrett, Shanthi Ameratunga, Suzanne Polinder, Ronan A Lyons, Frederick P Rivara, James E Harrison

**Affiliations:** Department of Epidemiology and Preventive Medicine, Monash University, Melbourne, Australia; Centre for Improvement of Population Health through E-records Research, Swansea University, Swansea, UK; Injury Prevention Research Unit, Department of Preventive and Social Medicine, Dunedin School of Medicine, University of Otago, Dunedin, New Zealand; School of Health and Social Services, Massey University, Palmerston North, New Zealand; Section of Epidemiology and Biostatistics, School of Population Health, University of Auckland, Auckland, New Zealand; Department of Public Health, Erasmus MC, Rotterdam, The Netherlands; Public Health Wales NHS Trust, Swansea, Wales UK; Departments of Pediatrics and Epidemiology, and the Harborview Injury Prevention and Research Center, University of Washington, Seattle, USA; Research Centre for Injury Studies, Flinders University, Adelaide, South Australia

**Keywords:** Injury, Agreement, SF-12, EQ-5D, Quality of life

## Abstract

**Background:**

Comparing health-related quality of life (HRQL) outcomes between studies is difficult due to the wide variety of instruments used. Comparing study outcomes and facilitating pooled data analyses requires valid “crosswalks” between HRQL instruments. Algorithms exist to map 12-item Short Form Health Survey (SF-12) responses to EQ-5D item responses and preference weights, but none have been validated in populations where disability is prevalent, such as injury.

**Methods:**

Data were extracted from the Validating and Improving injury Burden Estimates Study (Injury-VIBES) for 10,166 adult, hospitalized trauma patients, with both the three-level EQ-5D (EQ-5D-3L) and SF-12 data responses at six and 12-months postinjury. Agreement between actual (patient-reported) and estimated (mapped from SF-12) EQ-5D-3L item responses and preference weights was assessed using Kappa, Prevalence-Adjusted Bias-Adjusted Kappa statistics and Bland-Altman plots.

**Results:**

Moderate agreement was observed for usual activities, pain/discomfort, and anxiety/depression. Agreement was substantial for mobility and self-care items. The mean differences in preference weights were -0.024 and -0.012 at six and 12 months (p < 0.001), respectively. The Bland-Altman plot limits of agreement were large compared to the range of valid preference weight values (-0.56 to 1.00). Estimated EQ-5D-3L responses under-reported disability for all items except pain/discomfort.

**Conclusions:**

Caution should be taken when using EQ-5D-3L responses mapped from the SF-12 to describe patient outcomes or when undertaking economic evaluation, due to the underestimation of disability associated with mapped values. The findings from this study could be used to adjust expected EQ-5D-3L preference weights when estimated from SF-12 item responses when combining data from studies that use either instrument.

**Electronic supplementary material:**

The online version of this article (doi:10.1186/s12963-015-0047-z) contains supplementary material, which is available to authorized users.

## Introduction

Generic measures of health-related quality of life (HRQL) are recommended for burden and outcome studies, as they improve our understanding of patient-relevant physical, psychological, and social outcomes, allow comparison across different populations and interventions, and facilitate economic evaluations [1]. However, there is a multitude of generic measures of HRQL available for use worldwide, resulting in studies using a wide variety of instruments, with limited capacity to compare studies that use different measures [1]. Although a published consensus statement recommended use of the EQ-5D [2], Polinder et al’s review of the literature in 2010 acknowledged the use of 24 different generic measures of HRQL in injury outcome studies. Polinder et al recommended pooling data from individual studies to map or ‘crosswalk’ responses from different HRQL measures to gain a deeper understanding of injury outcomes [3].

While the 36-item Short Form Health Survey is arguably the most commonly used HRQL instrument in injury studies, both the EQ-5D and the 12-item Short Form Health Survey (SF-12) are also commonly used in injury populations [4–14]. The EQ-5D is brief, validated, and its empirically based community-derived social preference weights allow for economic analyses [15, 16]. The SF-12 is also a brief, validated instrument for measuring HRQL [17], but further mapping and conversion to the SF-6D is needed to use this instrument as a utility measure for economic evaluation [18].

Despite the prevalence of use of both instruments in injury studies, they are rarely used concurrently, limiting the opportunity to consider the equivalence of results obtained from one or the other measure. While published algorithms for mapping the SF-12 responses to the EQ-5D are available [16, 19, 20], these vary in complexity and the underlying statistical methods used. Published algorithms have been developed using population-representative data where the prevalence of disability is low, limiting the generalizability to populations with more severe health states [20]. No studies have investigated the validity of the SF-12 to EQ-5D mapping algorithms in an injury population. Validation of mapping algorithms is needed to establish whether the SF-12 can be crosswalked to the EQ-5D to compare outcomes of patients between studies and to facilitate pooled analyses of injury outcomes data. The aim of this study was to establish the level of agreement between EQ-5D item responses and preference weights mapped from SF-12 responses and EQ-5D item responses and preference weights measured directly from patient self-report in an injury population.

## Methods

### Setting

This study is part of the Validating and Improving injury Burden Estimates Study (Injury-VIBES). The Injury-VIBES project aims to provide improved methods for measuring the burden of nonfatal injury through analysis of pooled, de-identified, patient-level data from participants in six prospective cohort studies from Australia, New Zealand, the United Kingdom, the Netherlands, and USA [21].

### Datasets and participants

For the purposes of this study, Victorian State Trauma Registry (VSTR) and Victorian Orthopaedic Trauma Outcomes Registry (VOTOR) data were extracted from the Injury-VIBES dataset. These sources, unlike the other studies that have provided data for use in Injury-VIBES, collected both the EQ-5D and the SF-12. The VSTR is a population-based trauma registry that captures data about all major trauma patients in the state of Victoria (population 5.4 million) [22, 23]. The Injury-VIBES project included all hospitalized major trauma patients who met any of the following criteria: Injury Severity Score >15, admission to an intensive care unit for more than 24 h, or required urgent surgery [22]. The ISS is a measure of anatomical injury severity with an ISS > 15 commonly used to define major trauma [24, 25]. The VSTR defines urgent surgery as surgery within 24 h of injury involving intracranial, intrathoracic, or intra-abdominal operations, or fixation of pelvic or spinal fractures. Patients injured between January 2007 and March 2011 were included in the Injury-VIBES study [22]. The VOTOR is a sentinel site clinical registry that collects detailed data about all orthopaedic trauma cases admitted to hospital for more than 24 h. The VOTOR sites were chosen to represent multiple levels of trauma system care, with the detailed data collected at four hospitals used to inform orthopaedic care in Victoria, Australia and more widely [26]. For the Injury-VIBES study, any orthopaedic trauma patient meeting VSTR criteria was excluded from the VOTOR dataset to avoid multiple inclusions of the same patient in the analysis.

All adult (18 years and over) participants, admitted to hospital from March 2007 to March 2011, who had both EQ-5D observations and SF-12 data (which were administered at the same time and in the same order at each time point), were included in the analysis. Unlike the EQ-5D, there is no proxy version of the SF-12, and therefore, where the interview was not conducted directly with the patient (*e.g.*, cognitive issues due to traumatic brain injury or pre-existing conditions such as dementia), the SF-12 was not administered [23]. As this study required both EQ-5D and SF-12 responses, cases where the SF-12 was not able to be administered were excluded.

The VSTR and VOTOR use an opt-out consent process where all eligible patients are included on the registries and provided with a letter and brochure explaining the purpose of the registries, the data collected, and what the data are used for (including research). The brochure and letter include instructions for how to have their data removed from the registry if they wish to do so. The opt-off rates are less than 1.0 % for the VSTR and 1.5 % for VOTOR. Any patients who had opted-off from the registries were not included in the Injury-VIBES study and Injury-VIBES was approved by the Monash University Human Research Ethics Committee.

### Outcome measures

At six and 12 months post-injury, the three-level EQ-5D (EQ-5D-3L) and the SF-12 Version 1 were collected via telephone interview. The EQ-5D-3L measures HRQL using five items (mobility, self-care, usual activities, pain/discomfort, and anxiety/depression), with each item having three possible responses: no problems, some problems, and extreme problems [27]. Responses to the 12 items of the SF-12 were used to calculate Physical Component Summary (PCS-12) and Mental Component Summary (MCS-12) scores (0-100), where higher scores equate to better physical and mental function [17].

### SF-12 Version 1 to EQ-5D-3L map

The algorithm described by Gray et al was used to estimate patient EQ-5D-3L responses from SF-12 Version 1 responses [20]. The algorithm was developed using data from 12,967 participants in the 2000 Medical Expenditure Panel Survey (MEPS), a representative survey of US citizens aged 18 years and older [20]. This algorithm was selected as it allows direct mapping from SF-12 item responses to EQ-5D-3L item responses rather than mapping to utility scores only [16, 19, 28]. Further, the chosen algorithm used multinomial logit regression rather than ordinary least squares regression used in a previous study. The multinomial logit regression approach was considered preferable to the ordinary least squares approach, because the latter is predicated on the assumptions that preference weights are normally distributed and that the probability of a score of 1.0 (full health) is low, assumptions which are not appropriate given the substantial ceiling effects which have been reported for the EQ-5D-3L [20]. Tariffs or value sets need to be applied to the EQ-5D-3L responses to generate the preference weights. For this study, the UK value sets (or tariffs) were used to calculate EQ-5D-3L preference weights, as these are most commonly used [12, 27].

### Data analysis

Kappa statistics, unweighted and linear weighted, were used to describe the agreement between the estimated (mapped from SF-12) and the actual (direct patient report) individual items of the EQ-5D-3L. The weighted Kappa is an extension of a simple Kappa where less weight is assigned to large differences between ratings than to small differences [29]. Prevalence-Adjusted Bias-Adjusted Kappa (PABAK) statistics were calculated to account for the effect of bias and/or prevalence on Kappa estimates [30]. For example, if there is a low or high proportion of responses in a single category, the Kappa statistic will be influenced by the prevalence of ratings, resulting in the apparently paradoxical combination of high percentage agreement and a low Kappa value [31]. The Kappa statistic will be influenced by bias when there is imbalance in the direction of disagreements [29]. Stuart-Maxwell tests of marginal homogeneity were performed to identify unidirectional bias between the estimated and actual EQ-5D item responses, which would indicate the need for calculation of PABAK.

In the absence of a universally accepted guideline for interpreting Kappa coefficients [29], the Landis and Koch guideline was used [32], as this guideline is widely applied and considered acceptable for evaluating the magnitude of Kappa statistics [33]. Therefore, for all Kappa statistics, a value of <0 was interpreted as poor agreement, 0 to 0.20 slight agreement, 0.21 to 0.40 fair agreement, 0.41 to 0.60 moderate agreement, 0.61 to 0.80 substantial agreement, and 0.81 to 1.00 almost perfect agreement [32]. Ninety-five percent confidence intervals (95 % CI) of Kappa and PABAK were calculated using the 95^th^ percentile from 200 bootstrap replications.

Bland-Altman plots were generated to plot the difference between actual and estimated EQ-5D-3L preference weights against the mean of the actual and estimated preference weights. The mean difference provides the estimate of bias while the limits of agreement provide an estimate of the influence of random variation [34]. A Wilcoxon signed-rank test was used to test whether actual and estimated EQ-5D preference weights differed. Analyses were performed using Stata 13.1 (StataCorp Inc., College Station, Texas).

## Results

A total of 10,166 patients were included in the study; 6060 patients had data at both time points, 1444 had data at six months but not 12 months, and 2662 had data at 12 months but not six months. There were 6377 (63 %) males and 3789 (37 %) females with a mean (SD) age of 47.6 (20.3) years (Table 1). Falls and road trauma were the predominant causes of injury (Table 1).

**Table 1:** Characteristics of study participants

Population descriptor	
Age (mean(SD) years)	47.6 (20.3)
Gender (n = 10,166), N (%)	6377 (62.7)
Male	3789 (37.3)
Female	2964 (29.8)
Cause of injury (n = 9960), N (%)	1531 (15.4)
Low fall (≤1 meter)	1413 (14.2)
Motor vehicle crash	1201 (12.0)
High fall (>1 meter)	650 (6.5)
Motorcycle crash	389 (3.9)
Pedal cyclist crash	1812 (18.2)
Pedestrian incident	
Other	
Some and severe problems with EQ-5D items at 6 months (n = 7504), N (%)	
Mobility	3024 (40.3)
Self-care	1093 (14.6)
Usual activities	3902 (52.0)
Pain/discomfort	4332 (57.7)
Anxiety/depression	2514 (33.5)
Some and severe problems with EQ-5D items at 12 months (n = 8722), N (%)	
Mobility	2968 (34.0)
Self-care	1089 (12.5)
Usual activities	3867 (44.3)
Pain/discomfort	4323 (49.6)
Anxiety/depression	2688 (30.8)
6 months (n = 7504)	
Mean (SD) PCS-12^a^	41.9 (12.1)
Mean (SD) MCS-12^b^	52.0 (11.0)
Mean (SD) EQ-5D-3L preference weight	0.72 (0.28)
12 months (n = 8722)	
Mean (SD) PCS-12	43.9 (12.3)
Mean (SD) MCS-12	52.0 (10.8)
Mean (SD) EQ-5D-3L preference weight	0.75 (0.28)

^a^ PCS-12, Physical Component Summary score of SF-12; ^b^ MCS-12, Mental Component Summary score of SF-12

Table 2 shows the frequency of patient-reported responses to each item of the EQ-5D-3L versus the estimated EQ-5D-3L responses from the SF-12. The number (Table 2) and percentage (Table 3) of cases in which the estimated EQ-5D-3L responses under-reported the level of disability was higher for all items except pain/discomfort. Unidirectional bias for all items at both time points was observed (Table 3). Under-estimation of disability was most notable for the usual activities and self-care items (Table 3). Overall, agreement between estimated EQ-5D-3L individual items and actual item responses ranged from 65 % for pain/discomfort at six months to 86 % for self-care at 12 months (Table 3).

**Table 2:** Actual EQ-5D-3L versus estimated (using SF-12) EQ-5D-3L responses for each EQ-5D-3L item*

		Estimated EQ-5D-3L responses from SF-12		
		No problems	Some problems	Severe problems
Actual EQ-5D-3L responses				
6 months	(n = 7504)			
Mobility	No problems	3756	715	9
	Some problems	1177	1774	35
	Severe problems	8	29	1
Self-care	No problems	6094	281	36
	Some problems	828	193	18
	Severe problems	40	12	2
Usual activities	No problems	3143	436	23
	Some problems	1642	1781	188
	Severe problems	49	174	68
Pain/discomfort	No problems	2190	953	29
	Some problems	966	2462	382
	Severe problems	31	268	223
Anxiety/depression	No problems	4310	647	33
	Some problems	868	1075	146
	Severe problems	74	219	132
12 months	(n = 8722)			
Mobility	No problems	4941	804	9
	Some problems	1154	1736	40
	Severe problems	17	21	0
Self-care	No problems	7318	287	28
	Some problems	829	187	27
	Severe problems	31	13	2
Usual activities	No problems	4333	496	26
	Some problems	1611	1785	249
	Severe problems	38	138	46
Pain/discomfort	No problems	3185	1180	34
	Some problems	916	2467	384
	Severe problems	22	282	252
Anxiety/depression	No problems	5294	699	41
	Some problems	913	1102	155
	Severe problems	91	278	149

*No shading represents agreement, light shading represents over-estimation, and darker shading represents under-estimation of problems by the SF-12 to EQ-5D-3L algorithm

**Table 3:** Agreement between actual components and estimated (using SF-12) components of EQ-5D-3L

EQ-5D-3L items	% agreement	% under-estimated	% over-estimated	Kappa (95 % CI)	Test of symmetry	PABAK*(95 % CI)
6 months (n = 7504)						
Mobility	73.7	16.2	10.1	0.44 (0.42, 0.47)	<0.001	0.61 (0.59, 0.62)
Self-care	83.8	11.7	4.5	0.18 (0.15, 0.21)	<0.001	0.76 (0.74, 0.77)
Usual activities	66.5	24.9	8.6	0.38 (0.36, 0.40)	<0.001	0.50 (0.48, 0.51)
Pain/discomfort	65.0	16.9	18.2	0.38 (0.36, 0.40)	<0.001	0.47 (0.46, 0.49)
Anxiety/depression	73.5	15.5	11.0	0.42 (0.41, 0.44)	<0.001	0.60 (0.59, 0.62)
12 months (n = 8722)						
Mobility	76.6	13.7	9.8	0.47 (0.45, 0.49)	<0.001	0.65 (0.63, 0.66)
Self-care	86.1	10.0	3.9	0.19 (0.17, 0.22)	<0.001	0.79 (0.78, 0.80)
Usual activities	70.7	20.5	8.8	0.42 (0.40, 0.43)	<0.001	0.56 (0.55, 0.57)
Pain/discomfort	67.7	14.0	18.3	0.43 (0.41, 0.44)	<0.001	0.52 (0.50, 0.53)
Anxiety/depression	75.0	14.7	10.3	0.43 (0.41, 0.45)	<0.001	0.63 (0.61, 0.64)

*Prevalence –adjusted Bias-adjusted Kappa

Without adjustment for prevalence or bias, the Kappa statistics suggested fair to moderate agreement for all items, except for self-care where agreement was slight. Weighted Kappa statistics were calculated but differed little from the unweighted Kappas (results not shown). However, the prevalence of actual severe problems was low for several EQ-5D-3L items, and unidirectional bias was evident, suggesting the need to account for prevalence and bias. Therefore, the PABAK results are shown in Table 3. After accounting for prevalence and bias, the level of agreement ranged from moderate for usual activities, pain/discomfort, and anxiety/depression to substantial for the mobility and self-care items (Table 3). Kappa and PABAK statistics were calculated for VOTOR and VSTR to explore any differences in agreement related to severity of injury (*i.e.*, orthopedic trauma admission excluding major trauma versus major trauma). The results are shown in the Additional file 1: Table S1, and show comparable levels of agreement, except for higher PABAK agreement levels for the anxiety or depression item for VOTOR cases compared to VSTR cases.

**Fig. 1:**
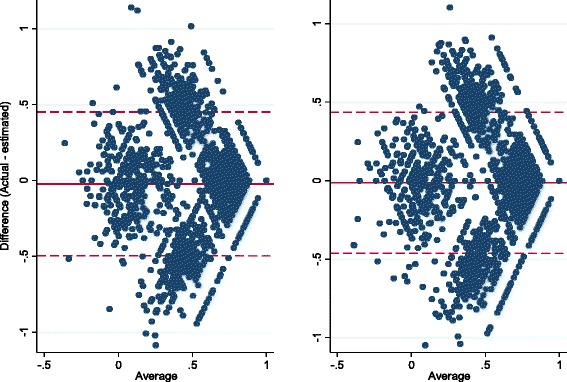
Bland-Altman Plot of actual, patient-reported EQ-5D-3L preference weights versus EQ-5D-3L preference weights estimated from SF-12 responses. Each marker represents one patient actual-estimated pair. The *x-*axis shows the mean of the patient-reported, actual, EQ-5D-3L preference weights, and the EQ-5D-3L preference weights estimated from SF-12 responses. The *y-*axis shows the difference between the actual and estimated preference weights. The *solid line* represents the overall mean difference between actual and estimated preference weights scores, and the *dashed lines* represent the limits of agreement (1.96 SD mean difference), which include 95 % of differences between actual and estimated responses. Where perfect agreement is observed, individual points line up along the 0 line of the y-axis

The mean difference between the actual and estimated EQ-5D preference weights at six and 12 months were -0.024 and -0.012, respectively (Table 4). The differences were small, but statistically significant, suggesting under-estimation of disability when using EQ-5D-3L preference weights mapped according to the method of Gray *et al.* [20]. The Bland-Altman plot showed relatively few points aligned along the horizontal zero line which would reflect perfect agreement (Fig. 1). There were many points outside the limits of agreement, and the limits of agreement were large compared to the range of valid EQ-5D-3L values (-0.56 to 1.00), showing wide variation in the agreement between the two methods.

**Table 4:** Comparison of the actual EQ-5D-3L preference weight and the estimated EQ-5D preference weight

	Actual mean (SD)	Estimated mean (SD)	Z-score (*p*-value)	Mean difference (95 % CI)	Limits of agreement	Absolute difference (95 % CI)
6 months (n = 7504)	0.72 (0.28)	0.74 (0.28)	13.1 (<0.001)	−0.024 (-0.029, -0.019)	−0.498 to 0.450	0.154 (0.150, 0.158)
12 months (n = 8722)	0.75 (0.28)	0.76 (0.28)	7.6 (<0.001)	−0.012 (-0.017, -0.007)	−0.461 to 0.436	0.140 (0.138, 0.142]

## Discussion

Mapping algorithms, often described as “crosswalks” or bridging tables, can improve the capacity to compare outcomes between studies using different HRQL measures and enable pooled data analyses [3]. In this study, the estimated EQ-5D-3L, mapped from SF-12 item responses, consistently underestimated disability for four of the five EQ-5D-3L items compared to actual patient-reported responses in a population of hospitalized injury patients. The level of agreement, after accounting for the prevalence and bias in responses, was moderate for the usual activities, pain or discomfort, and anxiety or depression items and substantial for the mobility and self-care items. The mean preference weight based on patient-reported EQ-5D-3L responses was significantly lower than the mean EQ-5D-3L preference weight mapped from the SF-12 responses, confirming the under-estimation of disability noted in the individual item responses. However, the mean difference was small, reflecting the net effect of over-estimation of mapped disability for one item, under-estimation for the remaining items, and the very wide limits of agreement.

Algorithm developers reported declining performance of the mapping algorithm with poorer health states [20] and underestimation of poorer health status as issues, recommending further validation in populations with higher levels of disability [19]. In our study, the lower level of agreement observed between the mapped and actual EQ-5D-3L responses is likely to reflect the higher prevalence of poorer health status in our injury population compared to the general population. The 12-month mean EQ-5D-3L preference weight, PCS-12, and MCS-12 were 0.75, 43.9, and 52.0, respectively, compared to 0.82, 49.5, and 51.4 in the MEPS sample used to develop the algorithm [20], and 3.3 % of our cases recorded EQ-5D-3L preference weight scores considered worse than being dead, compared to 1.4 % of the MEPS sample [19]. The observed levels of agreement were similar for the VSTR and the VOTOR datasets (Additional file 1: Table S1) despite the difference in the injury severity and age profile of these studies, where the VSTR includes major trauma patients only who tend to be younger, and the VOTOR subset includes an older population of only orthopaedic trauma admissions that do not meet major trauma criteria. The findings are likely to be similar as both increasing age and severity would result in poorer HRQL.

A strength of the study was the large sample size. Both HRQL measures were collected at two time points, six and 12 months postinjury. However, the study was limited to patients from one health care setting in Australia (population 5.4 M), and was also limited to hospitalized patients only. It is possible that the findings may not generalize to less severely injured populations or those from other health care contexts, such as primary care and emergency department presentation. It is possible that in less severely injured patients, where persisting disability would be expected to be less prevalent and milder, the algorithms may perform better. However, it should be noted that studies of non-hospitalized injured patients also report relatively high prevalence of persisting disability [35]. Cognitive deficits after traumatic brain injury and pre-existing conditions such as dementia often preclude direct interview of patients. No version of the SF-12 has been endorsed for administration via a proxy subject and so these patients in VSTR and VOTOR lack SF-12 data and could not be included in this analysis. The findings of this study therefore reflect an injury population without serious cognitive limitations. However, it should be noted that this would be a limitation for any study involving mapping of SF-12 to EQ-5D.

## Conclusions

Overall, we found moderate to substantial agreement between actual EQ-5D-3L responses and mapped responses from the SF-12 in an injured population after accounting for prevalence and bias in responses. EQ-5D-3L item responses estimated from SF-12 responses under-estimated disability for most items when compared to EQ-5D-3L responses collected directly from patients. While the mean difference between actual and estimated EQ-5D-3L preference weights was small, under-estimation of disability was evident. Caution should be taken when using mapped data to describe patient outcomes or when using mapped EQ-5D-3L responses for economic evaluation, due to the underestimation of disability associated with mapped values, particularly if using individual EQ-5D items. Nevertheless, the findings from this study could be used to adjust expected EQ-5D preference weights when estimated from SF-12 item responses. Finally, similar validation of these mapping algorithms is warranted in other health condition populations, particularly where the prevalence of disability is high.

## Additional file

Additional file 1: Table S1. Kappa statistics stratified by source of data.

## Abbreviations

HRQL: Health related quality of life
SF-12: 12-item Short Form Health Survey
EQ-5D-3L: Three-level EQ-5D
Injury-VIBES: Validating and Improving injury Burden Estimates Study (Injury-VIBES)
VSTR: Victorian State Trauma Registry
VOTOR: Victorian Orthopaedic Trauma Outcomes Registry
PCS-12: Physical Component Summary score of the SF-12
MCS-12: Mental Component Summary score of the SF-12
PABAK: Prevalence-Adjusted Bias-Adjusted Kappa
MEPS: Medical Expenditure Panel Survey
