# Supplementary material for: Level of agreement between patient-reported EQ-5D responses and EQ-5D responses mapped from the SF-12 in an injury population
Source: Popul Health Metr. 2015 Jun 13;13:14. doi: 10.1186/s12963-015-0047-z (PMC4474565; doi:10.1186/s12963-015-0047-z)
Supplement: Additional file 1: Table S1. — Kappa statistics stratified by source of data. [file 12963_2015_47_MOESM1_ESM.pdf]

Supplementary Table: Kappa statistics stratified by source of data

EQ-5D item	VSTR** Kappa (95% CI)	VOTOR Kappa (95% CI)	VSTR PABAK* (95% CI)	VOTOR PABAK (95% CI)
**6 months**	**N = 2394**	**N = 5110**	**N = 2394**	**N = 5110**
Mobility	0.46 (0.43, 0.50)	0.44 (0.41, 0.46)	0.62 (0.59, 0.64)	0.60 (0.58, 0.62)
Self-care	0.22 (0.17, 0.27)	0.16 (0.13, 0.20)	0.74 (0.71, 0.76)	0.77 (0.75, 0.78)
Usual activities	0.42 (0.39, 0.45)	0.35 (0.33, 0.38)	0.52 (0.49, 0.54)	0.49 (0.47, 0.51)
Pain/discomfort	0.41 (0.38, 0.44)	0.36 (0.34, 0.39)	0.49 (0.46, 0.52)	0.47 (0.45, 0.49)
Anxiety/depression	0.44 (0.41, 0.48)	0.41 (0.38, 0.43)	0.57 (0.54, 0.59)	0.62 (0.60, 0.64)
**12 months**	**N = 2846**	**N = 5876**	**N = 2846**	**N = 5876**
Mobility	0.45 (0.42, 0.49)	0.47 (0.45, 0.49)	0.63 (0.60, 0.65)	0.66 (0.64, 0.67)
Self-care	0.23 (0.18, 0.27)	0.17 (0.13, 0.20)	0.76 (0.74, 0.78)	0.81 (0.79, 0.82)
Usual activities	0.43 (0.40, 0.46)	0.40 (0.38, 0.43)	0.55 (0.53, 0.58)	0.56 (0.55, 0.58)
Pain/discomfort	0.43 (0.40, 0.46)	0.42 (0.40, 0.44)	0.51 (0.48, 0.54)	0.52 (0.50, 0.54)
Anxiety/depression	0.42 (0.39, 0.45)	0.43 (0.41, 0.46)	0.56 (0.53, 0.58)	0.66 (0.64, 0.67)

*VSTR, Victorian State Trauma Registry; **VOTOR, Victorian Orthopaedic Trauma Outcomes Registry; ***PABAK, Prevalence And Bias Adjusted Kappa
